# Masson pine pollen aqueous extract ameliorates cadmium-induced kidney damage in rats

**DOI:** 10.3389/fmolb.2023.1249744

**Published:** 2023-12-06

**Authors:** Zhiyong Hu, Sixin Chen, Tala Shi, Zhaoju Dong, Mei Cheng, Ning Li, Huijuan Zhao, Haibo Zhu, Chunlei Han, Lanlan Xu

**Affiliations:** ^1^ Department of Occupational Health and Environmental Hygiene, School of Public Health and Management, Binzhou Medical University, Yantai, China; ^2^ School of Public Health and Management, Binzhou Medical University, Yantai, China; ^3^ Department of Health and Disease Management, Binzhou Medical University, Yantai, China

**Keywords:** Masson pine pollen, cadmium, transcriptome, metabolome, conjoint

## Abstract

**Introduction:** Cadmium (Cd) is a hazardous environmental pollutant present in soil, water, and food. Accumulation of Cd in organisms can cause systematic injury and damage to the kidney. The Masson pine pollen aqueous extract (MPPAE) has attracted increasing attention due to its antioxidant activity and ability to enhance immunity.

**Methods:** In this study, we investigated the potential of MPPAE to protect against Cd-induced kidney damage in rats and the underlying mechanism. The transcriptome and metabolome of rats with Cd-induced kidney damage, following treatment with MPPAE, were explored.

**Results:** The concentrations of superoxide dismutase (SOD) and malondialdehyde (MDA) were both significantly altered after treatment with MPPAE. Furthermore, sequencing and analysis of the transcriptome and metabolome of rats with Cd-induced kidney damage, following treatment with MPPAE, revealed differential expression of numerous genes and metabolites compared with the untreated control rats. These differentially expressed genes (DEGs) included detoxification-related genes such as cytochrome P450 and the transporter. The differentially expressed metabolites (DEMs) included 4-hydroxybenzoic acid, L-ascorbate, and ciliatine. Conjoint transcriptome and metabolome analysis showed that several DEGs were correlated with DEMs.

**Conclusion:** These preliminary findings indicate the potential of MPPAE for the treatment of toxic metal poisoning.

## Graphical Abstract

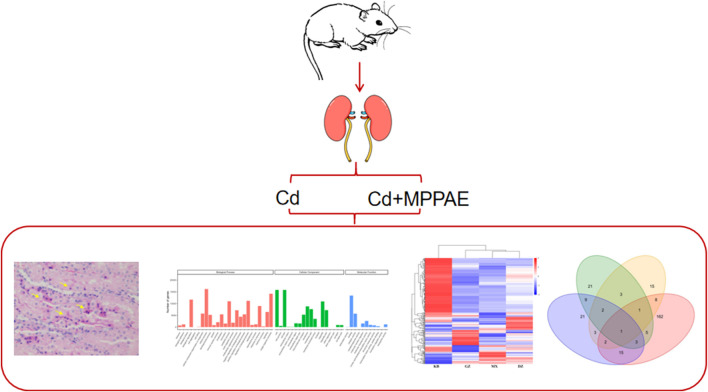

## 1 Introduction

The development of metallurgy, as well as the electroplating and chemical industries, has seen an increase in the release of cadmium into the environment, leading to Cd pollution (Rodrigues et al., 2020; Geng et al., 2022). Cd and its compounds enter the human body mainly through air, food, smoking, and drinking (II’yasova and Schwartz, 2005; Rinaldi et al., 2017). Subsequently, Cd accumulates and is distributed throughout the body organs via blood circulation, and finally causes systematic injury and damage to multiple organs including the bone, liver, kidney, and reproductive system (Horiguchi et al., 2011; Gomes de Moura and Ribeiro, 2017). Epidemiological investigations have shown that Cd exposure is closely associated with bone loss and osteoporosis (Luo et al., 2021; Wang et al., 2021). Furthermore, individuals with long-term exposure to low doses of Cd can develop respiratory symptoms, such as cough, increased sputum production, and asthma (Oh et al., 2014). Moreover, Cd could induce a serious kidney injury. An in-depth study found the mechanism by which Cd causes kidney injury. Yan et al. found that Cd could induce reactive oxygen species by impairing the function of NADPH oxidase, leading to oxidative damage and finally resulting in a decline in kidney function. Lemaire et al. (2020) found that Cd-induced kidney damage might be caused by dysregulated miRNAs (Yan and Allen, 2021). It is predicted that exposure of the general population to Cd will increase over the next few decades (Nawrot et al., 2010; Mezynska and Brzóska, 2018). Therefore, investigations to identify effective methods for decreasing the effects of Cd poisoning are very important.

At present, patients with Cd poisoning usually use traditional chelation therapy combined with new types of chelating agents and nanoparticle antidotes (Gil et al., 2011; Rafati R et al., 2017). However, the effect of this method is not ideal, and side effects can occur. Some studies have shown that selenium can be used as an antagonist of the toxicity caused by Cd, although this approach is limited by the similarity of the toxic and therapeutic doses of selenium (Branca et al., 2018; Xue et al., 2021).

Recently, plant extracts have attracted increasing attention for treating the effects of toxic metal poisoning due to their low incidence of side effects (Gurel et al., 2007). *In vivo* experiments showed that aqueous extracts from *Corchorus olitorius* L. leaves can downregulate the increased levels of glutamic–pyruvic transaminase and aspartate aminotransferase in blood induced by cadmium chloride (CdCl_2_) (4 mg/kg/d) treatment (Saikat et al., 2013). Ghosh et al. (2010) confirmed that fruit extracts of *Terminalia arjuna* had similar effects on the increased levels of glutamic–pyruvic transaminase and aspartate aminotransferase induced in mouse blood by Cd accumulation. Curcumin treatment protected mouse kidney tissues against the toxic effects of Cd, with no cell shrinkage and a significant reduction in the cavitation phenomenon after the injection of Cd (Gong et al., 2015). Similarly, compared with the control, the ethanol extract of Masson pine improved the relative cell survival rate after administration of Cd (Yuan et al., 2018). Moreover, epigallocatechin-3-gallate (EGCG), a green tea polyphenol, could mediate the biofabrication of nanoscale TiO_2_ as a function of pH. The EGCG corona plays important roles in enhancing the cell interaction by preventing agglomeration and dissolution, contributing to the penetration of mammalian tissues while encompassing complexed nano-bio-interfaces (Ali et al., 2023).

*Pinus massoniana* pollen, which is the traditional Chinese medicine food homology variety, is rich in many types of compounds, including polysaccharides, phytosterol, essential amino acids, and total polyphenol, with antioxidant activity and the ability to enhance immunity (Wei et al., 2011; Zhao et al., 2013; Yang et al., 2015). In an *in vitro* mechanistic study, Bao et al. (2006) reported that 100–600 μg/ml pine pollen aqueous extract functioned as an antioxidant by inhibiting myeloperoxidase activity, as well as eliminating reactive oxygen species, such as super oxygen free radicals, hydroxyl radicals, and hypochlorous acids. Lee et al. (2009) confirmed the antioxidant and inflammatory activities of the pine pollen extract *in vitro*.

In this study, we investigated the potential of the Masson pine pollen aqueous extract (MPPAE) to protect against Cd-induced kidney damage in rats and the underlying mechanism by adopting a combined transcriptomics and metabolomics approach. The differentially expressed genes (DEGs) and differentially expressed metabolites (DEMs) were identified, and their correlation was also investigated. The study aims to explore the potential molecular mechanism via which MPPAE protects against Cd-induced kidney damage. This information will form the basis of further improvements in the application of plant extracts for the treatment of toxic metal poisoning.

## 2 Methods and materials

### 2.1 Experiment materials

Specific pathogen-free Sprague–Dawley male rats (age, 7 weeks; weight, 180–200 g) were obtained from Shandong International Biotechnology Park Development (China) under license number SYXK (Lu) 20180030. The rats were maintained at 0°C–26°C and at a relative humidity of 40%–70%. Sterling MPPAE was obtained from New Era Health Industry (Group) Co., Ltd. Then, 0.3, 0.9, and 2.7 g/kg MPPAE/rat was adjusted for further analysis. The materials used in this study are shown in Table 1.

**TABLE 1:** Animal grouping and intervention method.

Group	Mouse sex	Mouse number	Treatment	
			Intraperitoneal injection	Gavage g/kg/d
CK	Male	8	0.9% saline solution	Ultrapure water
MG	Male	10	(2 mg/(2 mg/kg/d)	Ultrapure water
ELG	Male	10	CdCl_2_ (2 mg/kg/d)	30 mg/mL MPPAE
LG	Male	10	CdCl_2_ (2 mg/kg/d)	90 mg/mL MPPAE
HG	Male	10	CdCl_2_ (2 mg/kg/d)	270 mg/mL MPPAE

Note: CK, negative control group; MG (model group), CdCl_2_ alone; ELG (extremely low group), 0.3 g/kg MPPAE; LG (low group), 0.9 g/kg MPPAE; HG (high group), 2.7 g/kg MPPAE.

### 2.2 Evaluation of the protective effect of MPPAE against kidney injury caused by Cd accumulation *in vivo*

To induce kidney damage, Sprague–Dawley rats received intraperitoneal injections of Cd (2 mg/kg/d) for 7 days (Zhang et al., 2017), and rats in the negative control group (CK) received 0.9% saline solution via the same route injection. The rats then received different concentrations of MPPAE (0.3, 0.9, and 2.7 g/kg, respectively) or ultrapure water orally by gavage. The concentrations of MPPAE used in this study were determined based on previous reports and combined with preliminary experiments (Cong et al., 2015). Five groups were set for the following experiment: CK: negative control group; MG (model group): Cd alone; ELG (extremely low group): 0.3 g/kg; LG (low group): 0.9 g/kg MPPAE; HG (high group): 2.7 g/kg MPPAE. Indexes of malondialdehyde (MDA) and superoxide dismutase (SOD) in the kidney were investigated (*n* = 6 per group).

At 7 days post-treatment, the rats were euthanized and the kidneys were collected. Renal pelvis and papillae tissues were fixed in 4% paraformaldehyde and embedded in paraffin wax, and sections (3 μm thickness) were prepared. The sections were then stained with hematoxylin and eosin (HE) for the evaluation of histopathology under a microscope (×400 magnification).

### 2.3 RNA extraction and transcriptomic sequencing

Based on the *in vivo* experiments, transcriptomic and metabolomic analyses were conducted in the following groups: CK, MG, LG, and HG. For each group, total RNA was extracted from approximately 400 mg of kidney tissues using TRIzol, according to the manufacturer’s instructions. The quality of total RNA was evaluated using an Agilent 2100 Bioanalyzer. Subsequently, mRNAs were enriched using magnetic Oligo (dT) beads, followed by cDNA synthesis, and 250–300-bp cDNA fragments were selected for PCR amplification to construct the cDNA library. After quality control, the cDNA library was sequenced using the Illumina NovaSeq 6000 sequencing platform.

### 2.4 Data processing

Raw sequencing data were filtered to remove reads that contained adapter sequences, N bases, and low-quality reads. The clean data were then mapped to the reference genome using HISAT2 software (Mortazavi et al., 2008). Novel transcripts were assembled using StringTie software (Pertea et al., 2015), and the transcripts were then annotated using the Pfam, SUPERFAMILY (El-Gebali et al., 2019), Gene Ontology (GO), and Kyoto Encyclopedia of Genes and Genomes (KEGG) databases (Chen et al., 2017).

### 2.5 Differentially expressed gene analysis

The fragments per kilobase of transcript per million base pairs sequenced (FPKM) method, which is considered to reflect the influence of the sequencing depth and gene length of fragments, is commonly used to evaluate gene expression levels in transcriptomic data analysis (Bray et al., 2016). DESeq2 software was used to screen DEGs, defined as those with a fold change in expression >1, and a *p*-value < 0.5.

### 2.6 Non-targeted metabolomics profiling

Kidney tissues (100 mg) were frozen in liquid nitrogen and ground before the addition of 500 μl of 80% methanol. The mixture was vortexed and placed on ice for 5 min before centrifugation at 15,000 × g for 20 min (4°C). Then, the methanol content in the supernatant was diluted to 53% and then centrifuged at 15,000 × g for 20 min (4°C). Finally, the supernatant was used for LC-MS analysis.

The chromatographic separation was performed using a Hypersil GOLD column (C18) at 40°C. The positive mode contained 0.1% formic acid as solvent A and methanol as solvent B. The negative mode consisted of 5 mM ammonium acetate as solvent A and methanol as solvent B. The mass spectrum scan ranged from 100 to 1,500 m/z, with other parameters set as follows: spray voltage: 3.5 kV, sheath gas flow rate: 35psi, auxiliary gas flow rate: 10 L/min, capillary temperature: 320°C, S-lens RF level: 60, auxiliary gas heater temperature: 350°C, polarity: positive and negative, and MS/MS: data-dependent scans.

All the raw data were processed using CD 3.1 search software, and the metabolites were identified and quantified through peak filtration, peak extraction, peak size quantitation, and finally, normalization. All the identified metabolites were annotated by the KEGG (https://www.genome.jp/kegg/pathway.html), HMDB (https://hmdb.ca/metabolites), and LIPID MAPS databases (http://www.lipidmaps.org/). The differences between metabolites detected in the two treatment groups were evaluated by *t*-tests, and those with variable importance in projection (VIP) > 1, *p* < 0.05, and fold change in levels >2 or <0.5 were regarded as significant DEMs.

### 2.7 Conjoint analysis of the transcriptome and metabolome

Conjoint analysis of the transcriptome and metabolome was performed to further reveal the post-transcriptional regulation of expressed genes. Correction analysis of the DEGs and DEMs was conducted based on Pearson coefficients. KEGG enrichment was evaluated to analyze the common pathways between DEGs and DEMs and clarify the main biochemical pathways and signal transduction pathways that link the DEGs and their corresponding DEMs.

### 2.8 Statistical analysis

Differences in the MDA and SOD levels in the kidneys between groups were analyzed using GraphPad Prism 7 software (Schneider et al., 2021). *p* < 0.05 was considered to indicate statistical significance.

## 3 Results

### 3.1 The protective effect of MPPAE

**FIGURE 1:**
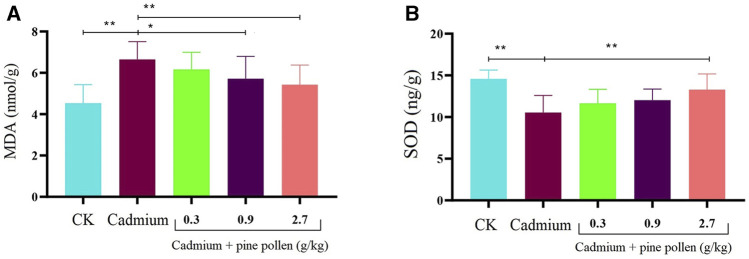
Influence of different concentrations of pine pollen on MDA (malondialdehyde) and SOD (superoxide dismutase) in the kidney. * means significant difference (*p* < 0.05), and ** represents extremely significant difference (*p* < 0.01). **(A)** Content of MDA; **(B)** content of SOD. Notes: CK: negative control group. The concentration of cadmium used in this study is 2 mg/kg/d. The concentrations of pine pollen used in this experiment are 0.3, 0.9, and 2.7. In panels A and B, the contents of MDA and SOD between the CK and cadmium injection group, cadmium injection group, and 2.7 g/kg pine pollen treatment group areextremely significantly different, respectively.

The kidney MDA contents of the MG Cd control group were significantly higher than those in the negative control group, while the SOD levels were significantly lower, indicating that the Cd-induced kidney injury model was successfully constructed in rats. After the application of MPPAE, the decrease in kidney MDA contents and the increase in SOD contents in the LG and HG groups were exacerbated. Furthermore, MPPAE at 0.9 and 2.7 g/kg exerted significant protection against Cd-induced kidney injury in a dosage-dependent manner. Therefore, model rats were treated with MPPAE at 0.9 and 2.7 g/kg for subsequent histopathology evaluation and transcriptomic and metabolomic analyses (Figure 1).

**FIGURE 2:**
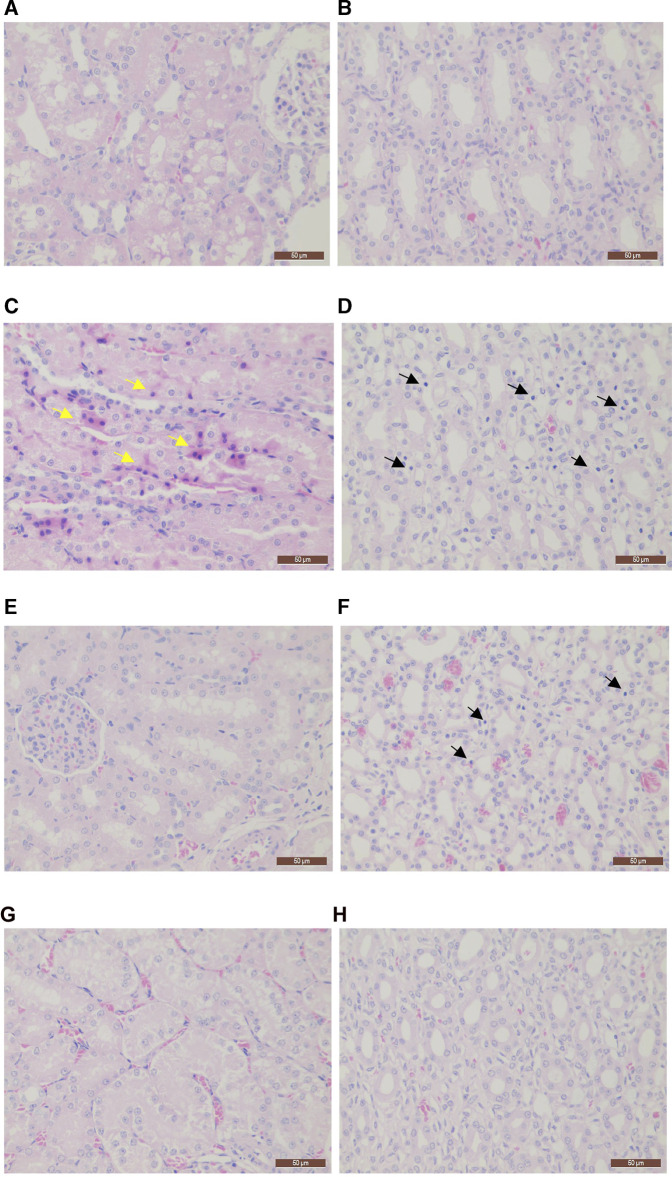
Histopathology sections (×400 magnification). **(A)** Cortex staining in the CK group; **(B)** renal papillae in the CK group; **(C)** cortex staining in the MG group; **(D)** renal papillae in the MG group; **(E)** cortex staining in the LG group; **(F)** renal papillae in the LG group; **(G)** cortex staining in the HG group; **(H)** renal papillae in the HG group. Note: CK: negative control group; MG (model group): CdCl_2_ alone; LG (low group): 0.9 g/kg MPPAE; HG (high group): 2.7 g/kg MPPAE. Yellow arrows indicate renal tubular epithelial cell degeneration; black arrows indicate renal papilla interstitial inflammatory cell infiltration.

After injection of Cd, the kidney exhibited marked changes characteristic of injury. In the MG group, the main lesion involved inflammatory cell infiltration of the renal papilla. Moreover, degeneration occurred in the cortex renal tubular epithelial cells with the frequency of 1/7 and in the renal tubular hyaline cast with the frequency of 3/7. Similar lesions were observed in the LG group. However, the frequency and severity of the lesions, especially the inflammatory reaction in the renal papilla, were significantly reduced compared to those in the MG group. In contrast, only one case of renal papilla mesenchymal inflammatory cell infiltration occurred in the HG group, and inflammatory cells were rare. Thus, our findings indicated that MPPAE protected against Cd-induced kidney injury in a dosage-dependent manner (Figure 2).

### 3.2 Transcriptomic data analysis

Transcriptomic sequencing yielded an average of 45,180,315 raw reads, from which an average of 6.68 G of clean data were filtered. Q20 in each group was higher than 97%, which indicated high-sequencing quality. In addition to the genes mapped to the reference genome, 2,031 novel transcripts were assembled. Finally, 34,914 genes were annotated in the Pfam database.

**FIGURE 3:**
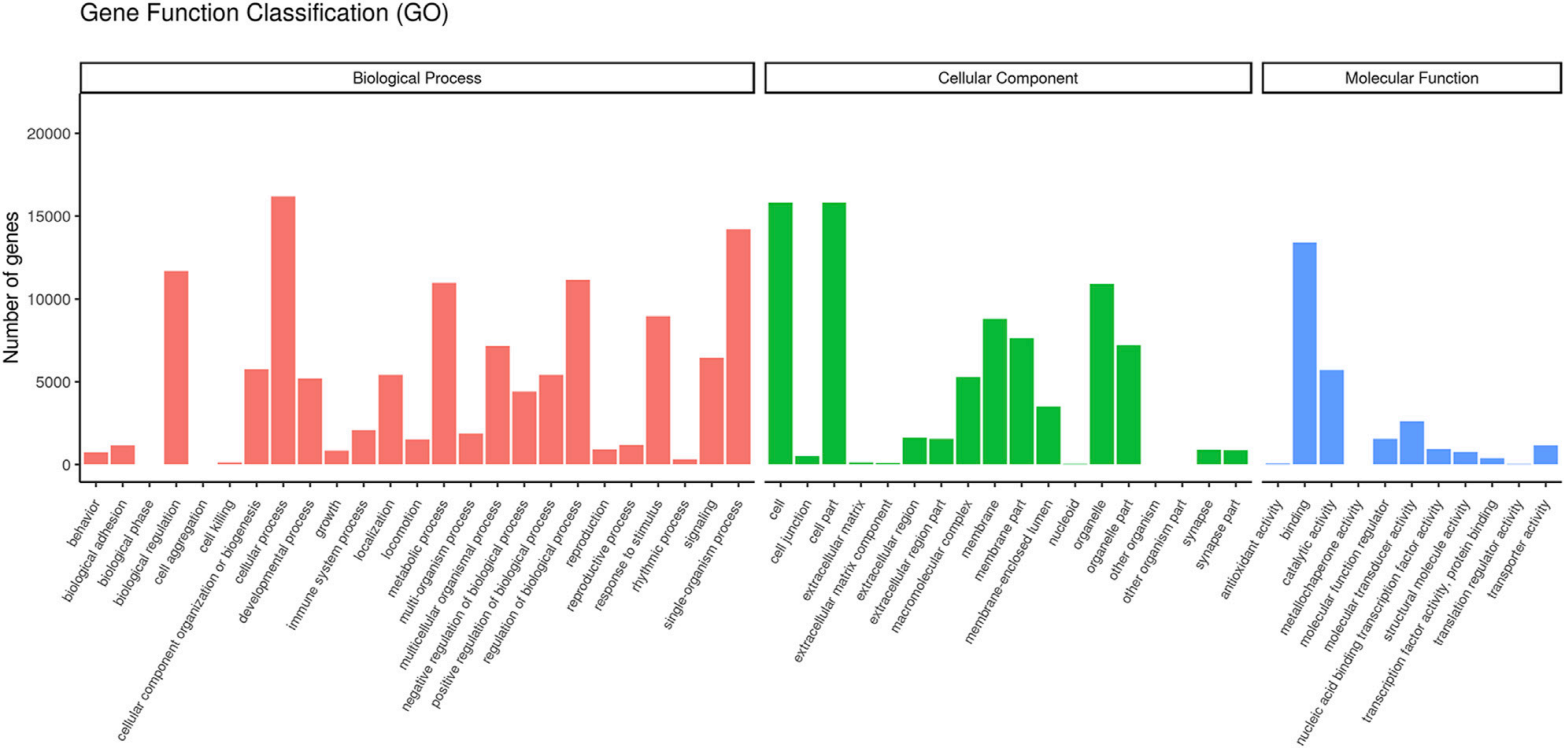
Gene Ontology function classification.

The GO classification showed that transcripts were enriched in three function categories of biological process, cellular component, and molecular function. Among these functions, cellular process (16,175 genes), cells (15,824 genes), and binding (13,404 genes) exhibit the highest proportion in each of these categories (Figure 3).

### 3.3 DEG analysis

**FIGURE 4:**
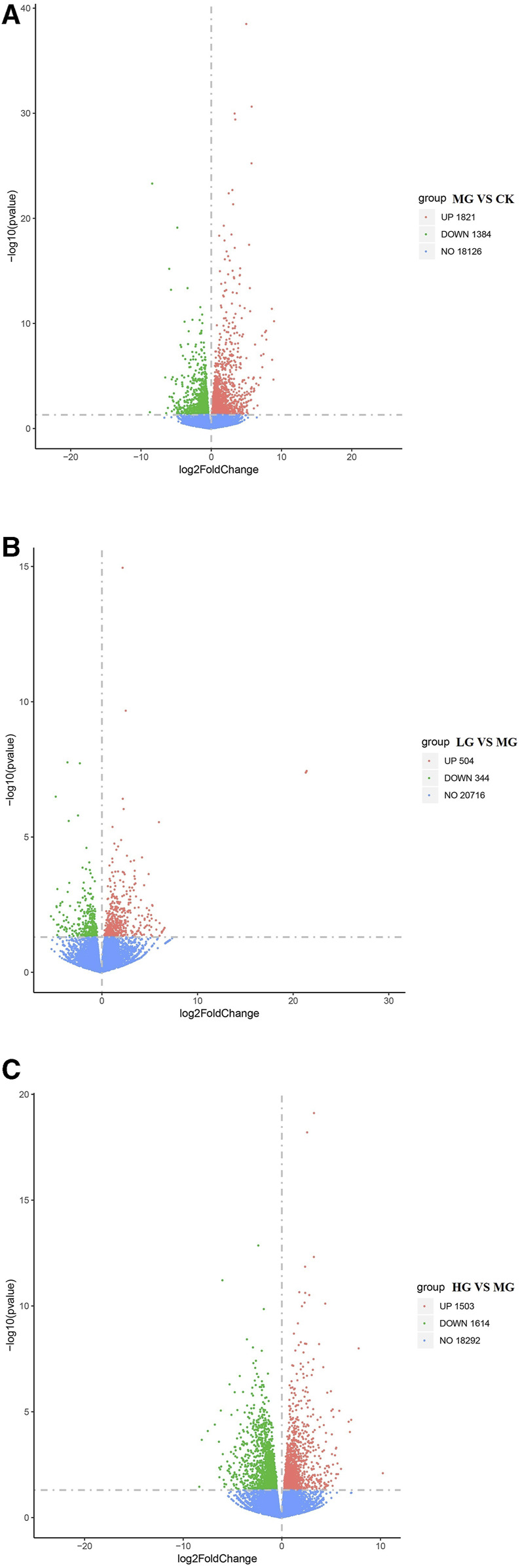
Differentially expressed genes in each treatment group. **(A)** MG vs. CK; **(B)** LG vs. MG; **(C)** HG vs. MG. Note: CK: negative control group; MG (model group): CdCl_2_ alone; LG (low group): 0.9 g/kg MPPAE; HG (high group): 2.7 g/kg MPPAE.

In comparison with the CK group, 3,205 DEGs (1,821 upregulated and 1,384 downregulated) were identified in the rats treated with Cd (Figure 4A), with the most significant changes observed in expression levels of genes encoding the L1 transposable element RBD-like domain, interleukin 1 receptor type 2, and cytochrome P450. In contrast, few DEGs (504 upregulated and 344 downregulated) were detected after treatment with the low concentration of MPPAE (Figure 4B), with the most significant changes observed in expression levels of genes encoding protein phosphatase 1, hypoxanthine phosphoribosyltransferase 1, and deoxyribonuclease 1-like 3. Compared with the MG group, 3,117 DEGs (1,503 upregulated and 1,614 downregulated) were identified in the rats treated with the high concentration of MPPAE (Figure 4C). The upregulated DEGs included several cytochrome P450-encoding genes and transporter-related genes. The number of common DEGs in both LG and HG groups compared with the MG group was 235, containing genes encoding cytochrome P450, zinc finger protein, ATP-binding cassette subfamily, *etc.*

### 3.4 Metabolite annotation

The detected metabolites were annotated to three important databases. Metabolites from both positive and negative ion detection modes exhibited similar annotation information in the KEGG database. The dominant pathways were global and overview maps, amino acid metabolism, and lipid metabolism. In the HMDB database, the number of metabolites and annotated superclasses was different in the positive or negative ion detection modes. The dominant annotated superclasses in the positive detection mode were lipids and lipid-like molecules, organic acids and derivatives, and organoheterocyclic compounds. In the negative detection mode, the dominant annotated superclasses were lipids and lipid-like molecules, organic acids and derivatives, and organic oxygen compounds.

In the LIPID MAPS database, there were significant differences in the annotation information between the positive or negative ion detection modes. In the positive ion detection mode, the dominant annotated classes were glycerophosphoethanolamines, steroids, and glycerophosphocholines. In the negative ion detection mode, the dominant annotated classes were fatty acids and conjugates, glycerophosphocholines, and eicosanoids.

### 3.5 Screening of DEMs

Numerous metabolites were differentially expressed after the injection of Cd. In the negative ion detection mode, 55 metabolites were upregulated after Cd and 77 were downregulated compared with the CK group. The most downregulated metabolite was 2-[(2-amino-6-methylpyrimidin-4-yl)thio]-4,6-dimethylnicotinonitrile, although no annotated information was available in any of the three databases, followed by hydroquinone. The most upregulated metabolites were sodium cholate and chenodeoxycholic acid. In the positive ion detection mode, 37 metabolites were upregulated and 160 were downregulated after the injection of Cd. The most upregulated metabolites were O-phospho-L-tyrosine and PC (20:5e/18:2). The most downregulated metabolites were tropolone and 2-(carboxymethoxyl)-4-methoxybenzoic acid.

Only minor changes in metabolites were observed after the application of low-dosage MPPAE. In the positive ion detection mode, 28 metabolites were upregulated, while only seven metabolites were downregulated. L-ascorbate and LPC 16:2 were the most upregulated metabolites, while prolyl leucine and ureidoisobutyric acid were the most downregulated. In the negative ion detection mode, 21 metabolites were upregulated, while only six were downregulated. The most upregulated metabolite was ciliatine, which was detected at 41.5-fold higher levels in the low-dosage MPPAE group compared with the control group without MPPAE treatment. The most downregulated metabolite was 4-hydroxy-3- methoxyphenylglycol sulfate.

Differences were detected in the degree of regulation of DEMs between the high- and low-dosage MPPAE groups. In the negative ion detection mode, 35 metabolites were upregulated, and the most differentially expressed metabolite was ciliatine. The levels of ciliatine in the high- and low-dosage MPPAE groups were 195.5-fold higher and 41.5-fold higher, respectively, than those in the control group without MPPAE treatment. These data indicated that ciliatine plays a crucial role in the mechanism by which MPPAE mediated the detoxification of Cd. The metabolites Dl-3,4-dihydroxymandelic acid and glyphosate were upregulated by 151.3-fold and 134.4-fold, respectively. In addition, 11 metabolites were downregulated, with D-glucuronic acid identified as the most differentially expressed metabolite. In the positive ion detection mode, 28 metabolites were upregulated and seven were downregulated.

**FIGURE 5:**
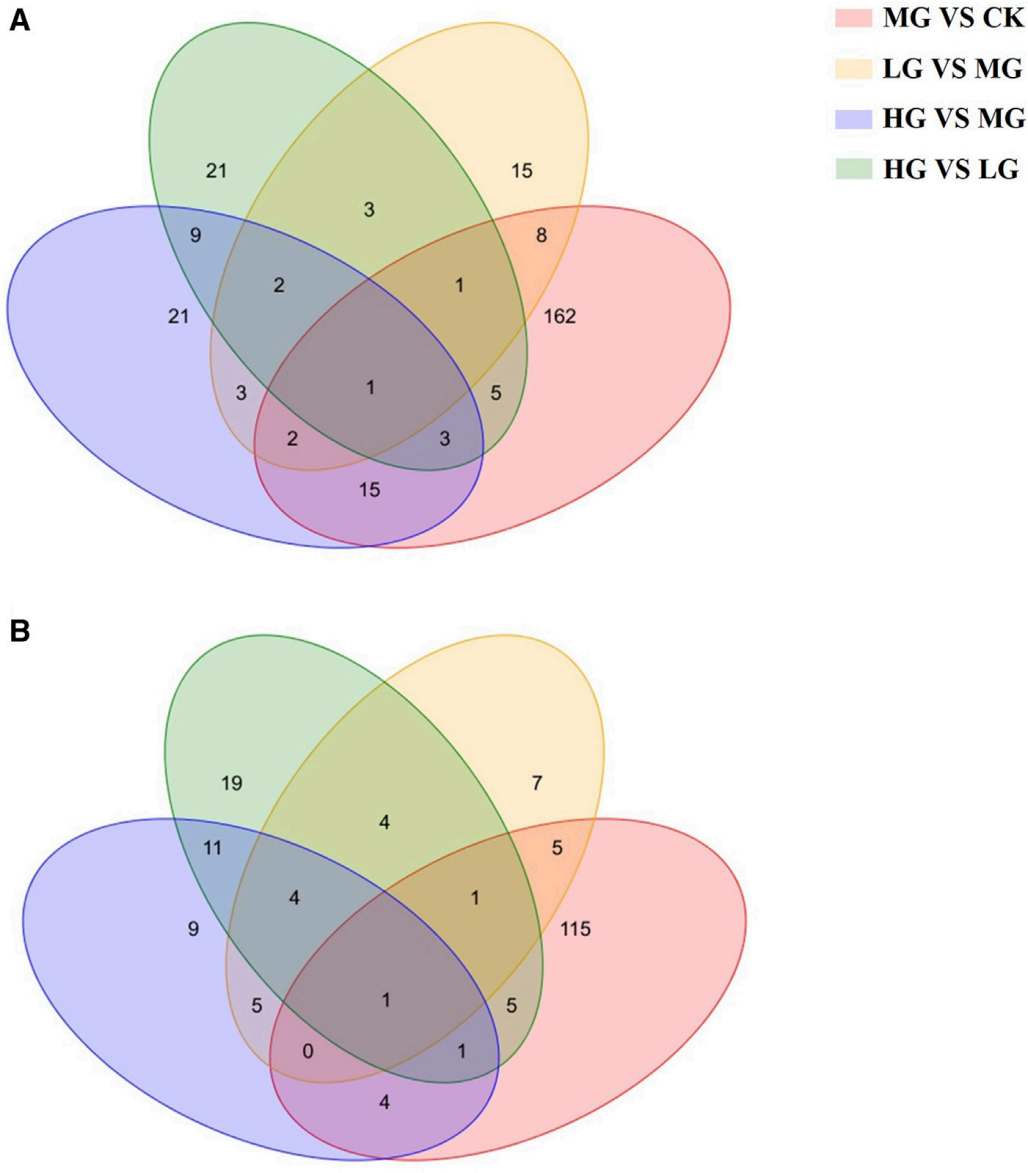
Venn analysis of overlapping and unique differentially expressed metabolites (DEMs) among the different treatment groups. **(A)**: positive ion detection mode; **(B)** negative ion detection mode. Note: CK: negative control group; MG (model group): CdCl_2_ alone; LG (low group): 0.9 g/kg MPPAE; HG (high group): 2.7 g/kg MPPAE.

Venn diagrams were generated to compare the overlapping and unique DEMs among the different treatment groups detected in the positive and negative ion detection modes (Figure 5). The number of common DEMs in both LG and HG groups compared with the MG group was 8 under the negative ion detection mode and 10 under the positive ion detection mode. One DEM, namely, 4-hydroxyretinoic acid, was detected under both negative and positive ion detection modes. From Venn diagram analysis, one DEM ascorbic acid and one DEM N1-(1,3-thiazol-2-yl)-2-chlorobenzamide were detected under negative and positive ion detection modes, respectively, among all groups. Hierarchical clustering analysis was also conducted to compare the metabolic changes under different treatments detected in the positive and negative ion detection modes.

### 3.6 KEGG enrichment analysis of DEMs

**FIGURE 6:**
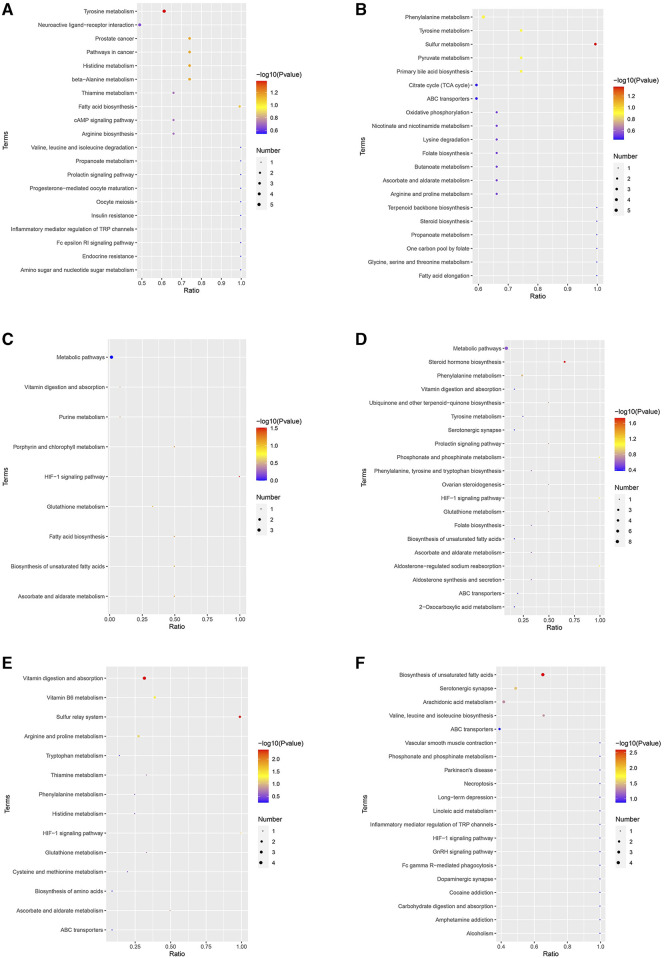
KEGG enrichment analysis of differentially expressed metabolites (DEMs). **(A)** MG vs. CK group in the positive ion detection mode; **(B)** MG vs. CK group in the negative ion detection mode; **(C)** LG vs. MG group in the positive ion detection mode; **(D)** LG vs. MG group in the negative ion detection mode; **(E)** HG vs. MG group in the positive ion detection mode; **(F)** HG vs. MG group in the negative ion detection mode. Note: CK: negative control group; MG (model group): CdCl_2_ alone; LG (low group): 0.9 g/kg MPPAE; HG (high group): 2.7 g/kg MPPAE.

After the injection of Cd, 41 and 50 KEGG pathways were enriched in the positive and negative ion detection modes, respectively. In the positive ion detection mode, the most enriched pathways were metabolic pathways and tyrosine metabolism, while in the negative ion detection mode, the most enriched pathways were metabolic pathways and phenylalanine metabolism (Figure 6). After the application of low-dosage MPPAE, nine and 22 KEGG pathways were enriched in the positive and negative ion detection modes, respectively, with metabolic pathways found to be dominant (Figure 6). Similarly, after the application of high-dosage MPPAE, metabolic pathways were dominant in both the negative (24 pathways) and positive (14 pathways) ion detection modes (Figure 6).

### 3.7 Conjoint analysis of the transcriptome and metabolome

**FIGURE 7:**
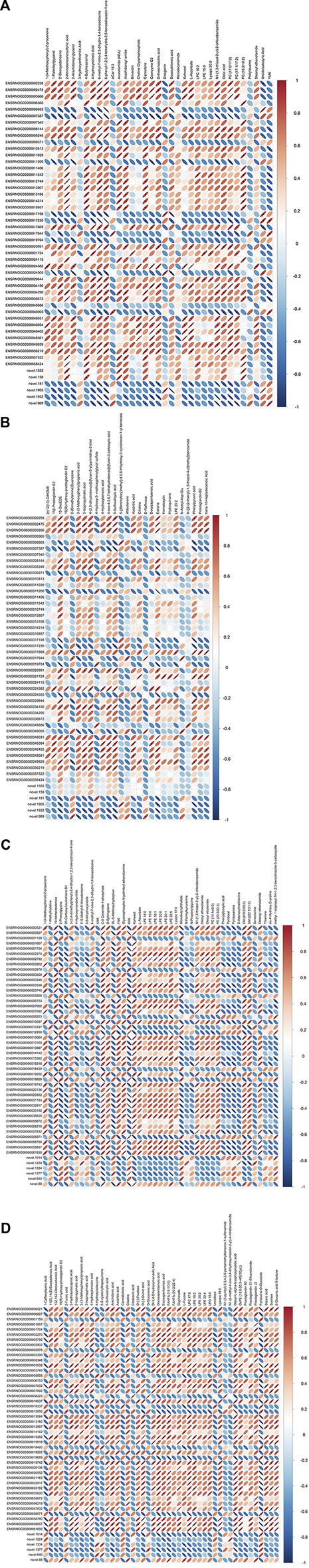
(Continued) Pearson correlation analysis of differentially expressed gene (DEG) and differentially expressed metabolite (DEM) coefficients. **(A)** LG vs. MG group in the positive ion detection mode; **(B)** LG vs. MG group in the negative ion detection mode; **(C)** HG vs. MG group in the positive ion detection mode; **(D)** HG vs. MG group in the negative ion detection mode. Note: CK, negative control group; MG (model group), CdCl_2_ alone; LG (low group), 0.9 g/kg MPPAE; HG (high group), 2.7 g/kg MPPAE.

Pearson correlation analysis of the DEGs and DEMs was conducted, with coefficients <0 representing a negative correlation, while correlation coefficients >0 represent a positive correlation (Figure 7).

**FIGURE 8:**
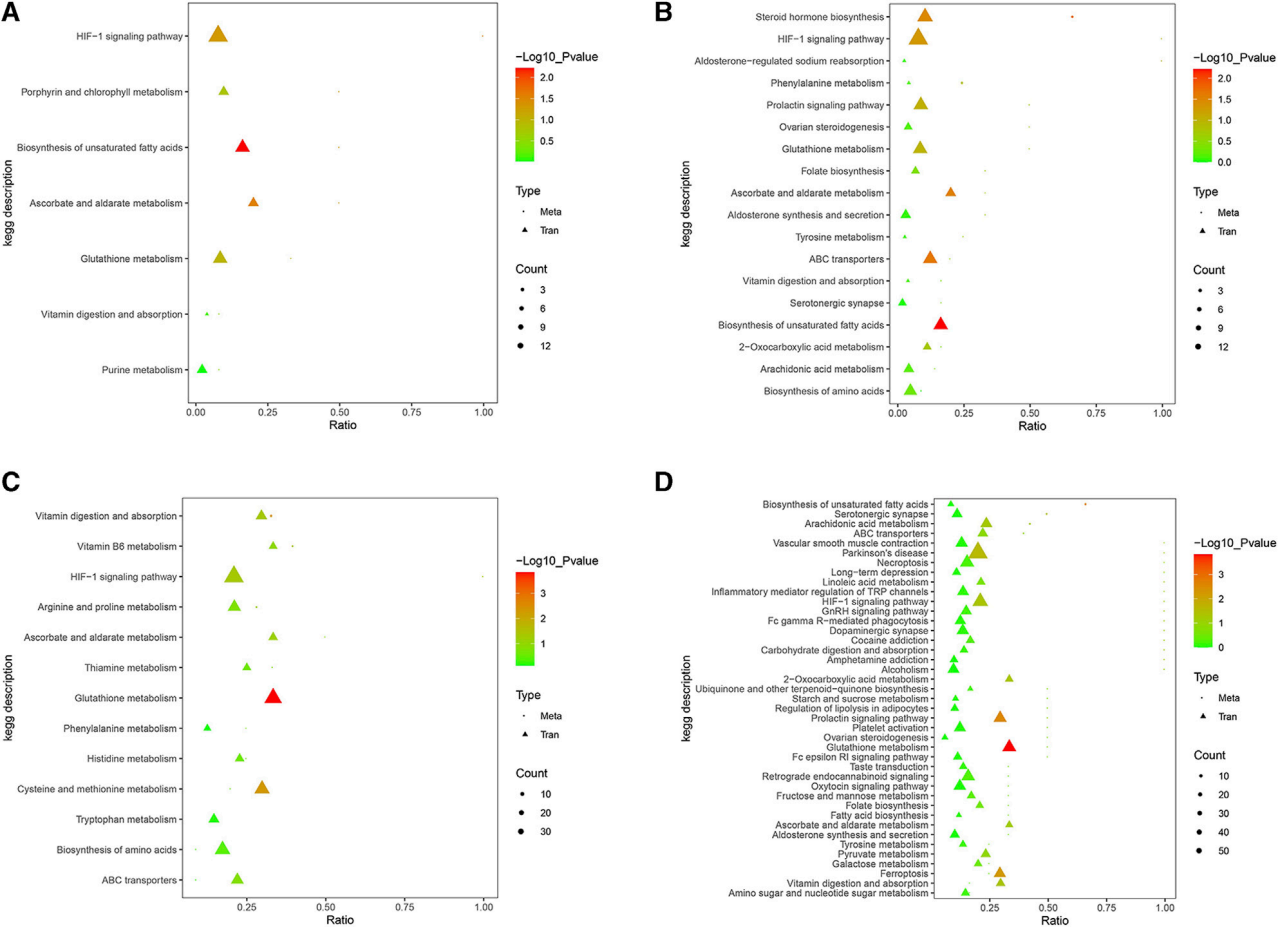
KEGG enrichment analysis differentially expressed gene (DEG) and differentially expressed metabolite (DEM) correlations. **(A)** LG vs. MG group in the positive ion detection mode; **(B)** LG vs. MG group in the negative ion detection mode; **(C)** HG vs. MG group in the positive ion detection mode; **(D)** HG vs. MG group in the negative ion detection mode. Note: CK, negative control group; MG (model group), CdCl_2_ alone; LG (low group), 0.9 g/kg MPPAE; HG (high group), 2.7 g/kg MPPAE.

KEGG enrichment analysis was conducted to identify the shared pathways associated with DEGs and DEMs, thereby clarifying their common biochemical pathways and signal transduction pathways. After the injection of Cd, 96 and 80 KEGG pathways were enriched in the positive and negative ion detection modes, respectively. After the application of low-dosage MPPAE, the enriched 16 KEGG pathways were enriched in the positive ion detection mode and 36 were enriched in the negative ion detection mode. In the low-dosage MPPAE treatment group, 26 KEGG pathways were enriched in the positive ion detection mode and 82 in the negative ion detection mode (Figure 8).

## 4 Discussion

Cd pollution can cause systematic injury and damage to multiple organs. Therefore, effective methods for reducing Cd toxicity are crucial. As an important plant extract, MPPAE has attracted increasing attention due to its antioxidant activity and the ability to enhance immunity. However, reports of the protective effect of MPPAE against Cd-induced kidney injury are rare. Therefore, it is of great significance to investigate the protective effects of MPPAE and the underlying mechanism. In this study, the protective effects of MPPAE against Cd-induced kidney injury were preliminarily verified. The DEGs and metabolites were then identified from transcriptomic and metabolomic analyses, and their correlation was also evaluated.

MDA and SOD are two important parameters that reflect the degree of cellular damage and antioxidant ability of cells, and can be used to evaluate the inhibition of inflammation (Wong et al., 2012; Islam et al., 2022). In this study, we found the kidney levels of MDA and SOD were significantly higher and lower, respectively, in rats after Cd administration compared with the levels detected in the blank control group, which indicated that Cd induced kidney damage. In contrast, the MDA and SOD contents in the groups treated with MPPAE were significantly lower and higher, respectively, than those in the Cd control group, which indicated that MPPAE protected rats against Cd-induced kidney damage, and functioned as an antioxidant to protect kidney cells in this model.

Histopathological analysis showed that Cd toxicity could result in inflammatory cell infiltration of the renal papilla, renal cortex tubular epithelial cell degeneration, and renal tubular hyaline cast formation. The application of MPPAE effectively alleviated the injury caused by Cd, with significantly reduced frequency and severity of lesions in the MPPAE treatment group, indicating that MPPAE protects against Cd-induced kidney injury. Sousa et al. found the activities of SOD could be stimulated by polyphenols, thereby strengthening the endogenous antioxidant system (Sousa et al., 2021). The MPPAE is a rich source of bioactive compounds that can play multiple rescue roles against the tissue damage. The infected Cd ions might be freely available to interact with the heterogeneous milieu of MPPAE polyphenols to decrease the tissue damage. A similar phenomenon has been found in green tea polyphenols, which could decrease oxidative DNA damage caused by exposure to metals. An in-depth study found that green tea polyphenols might directly eliminate free radicals, activate the repair oxidative DNA damage mechanism, or regulate the endogenous antioxidant system to decrease the DNA damage (García-Rodríguez et al., 2023). Therefore, we deduced that the polyphenols from MPPAE could stimulate the activities of SOD and further protect against Cd-induced kidney injury.

**FIGURE 9:**
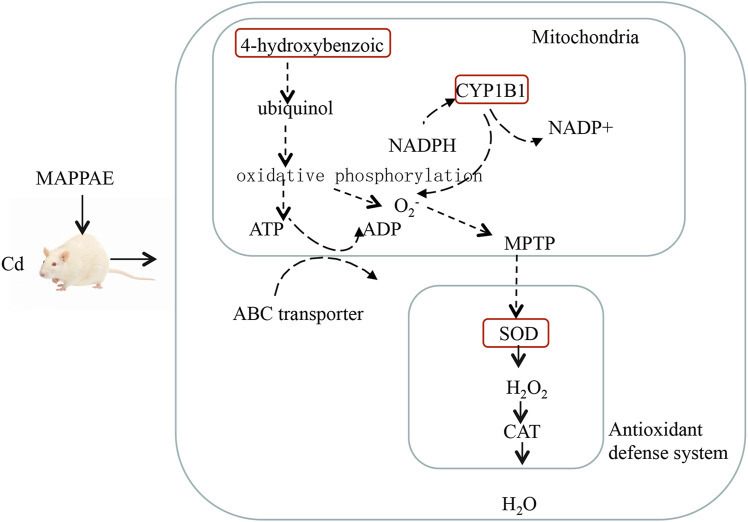
Potential mechanism presentation of the Cd ion-mediated interaction with kidneys and MPPAE.

Numerous DEGs were associated with the application of MPPAE. The upregulated DEGs included several cytochrome P450-encoding genes and transporter-related genes, which might be involved in the protection effect of MPPAE against Cd-induced kidney injury (Figure 9). Cytochrome P450, which belongs to the heme protein family, is known to play important roles in the metabolism of exogenous substances, such as drugs (Tomaszewski et al., 2008; Elfaki et al., 2018; Alzahran et al., 2020). Recent studies have shown that cytochrome P450 is related to detoxification. Bakheet et al. showed that the gene expression level of cytochrome P450 was significantly altered in humans exposed to toxic metals (Al Bakheet et al., 2013). Moreover, silencing of the cytochrome P450 gene reduced the ability to detoxify tannins (Zhao et al., 2022). Thus, the results of our study indicated that changes in the expression of genes encoding cytochrome P450 play a role in the mechanism underlying the Cd detoxification effects of MPPAE.

Transporters are membrane proteins involved in nutrient uptake, signal transduction, and metabolite release (Sasaki et al., 2016; Bröer, 2022). Recent studies have shown that transporter proteins are also associated with detoxification (Tamai and Tsuji, 2000; Orelle et al., 2018; Atkins, 2020). In this study, five transporter-related genes were upregulated in rats treated with MPPAE after the injection of Cd, indicating that transporter proteins are involved in the detoxification effect of MPPAE, possibly by mediating the extracellular transfer of the toxin. Further studies based on molecular techniques such as gene knockout and gene silencing are required to fully elucidate the function of selected DEG in the mechanism underlying the protective effects of MPPAE against Cd-induced kidney damage. Moreover, 235 DEGs, including cytochrome P450, zinc finger protein, and the ATP-binding cassette subfamily, were detected in both LG and HG groups compared with the MG group.

Investigating DEMs is also a very important approach to elucidating the mechanism by which MPPAE protects against Cd-induced kidney injury. In the metabolomic analysis, ciliatine was identified as a highly significant DEM associated with MPPAE treatment. Compared with the MG group, low-dosage MPPAE caused a 41.5-fold increase in ciliatine levels, and this increased to 195.5-fold upregulation in the high-dosage MPPAE group, indicating that ciliatine levels correlate positively with the dosage of MPPAE. Moreover, according to the conjoint analysis of the transcriptome and metabolomes, several DEGs encoding the ABC transporter subfamily were correlated with ciliatine, which is a component of the ABC transporter pathway. These findings indicate that ciliatine might play crucial roles in MPPAE detoxification, although the underlying mechanism remains to be elucidated.

Other metabolites related to stress tolerance, such as L-ascorbate, were also differentially expressed after the application of MPPAE. Compared with the MG group, low-dosage MPPAE caused a 2.9-fold upregulation of L-ascorbate, and this increased to a 15.9-fold upregulation, following treatment with high-dosage MPPAE, indicating mean L-ascorbate levels correlate positively with the dosage of MPPAE. L-ascorbate enriched the pathways of vitamin digestion and absorption, the HIF-1 signaling pathway, ascorbate and aldarate metabolism, and glutathione metabolism, and several DEGs were correlated with L-ascorbate. Ascorbic acid has been reported to be involved in plant stress tolerance conditions such as drought, extreme temperatures, and salt. The application of ascorbic acid improved the germination rate of alfalfa seeds and seedling length under 1.50% NaCl stress (Ou et al., 2022). In addition, 8 and 10 DEMs were detected under negative and positive ion detection modes, respectively, in both LG and HG groups compared with the MG group.

## 5 Conclusion

In this study, we showed that the concentration of antioxidant enzymes, such as SOD, was significantly altered after treatment with MPPAE. Furthermore, genes encoding proteins such as cytochrome P450 and the transporter known to be involved in detoxification processes were significantly differentially expressed under MPPAE treatment. Moreover, 4-hydroxybenzoic acid, L-ascorbate, and ciliatine were also identified as DEMs associated with MPPAE treatment. Moreover, several DEGs were correlated with DEMs through transcriptome and metabolome conjoint analysis. These preliminary findings indicate the potential of MPPAE for the treatment of toxic metal poisoning.

## Data Availability

The data were uploated to NCBI Sequence Read Archive (SRA) database with the accession numbers of SRR26952371, SRR26952372, SRR26836957, SRR26945012, SRR26912590, SRR26902607, SRR26870888, SRR26870777, SRR26870753, SRR26836530, SRR26836959 and SRR26836885.

## Abbreviation

DEMs, differentially expressed metabolites; DEGs, differentially expressed genes; EGCG, epigallocatechin-3-gallate; FPKM, fragments per kilobase of transcript per million base pairs sequenced; GO, Gene Ontology; HE, hematoxylin and eosin; KEGG, Kyoto Encyclopedia of Genes and Genomes; MPPAE, Masson pine pollen aqueous extract; MDA, malondialdehyde; SOD, superoxide dismutase; VIP, variable importance in projection.
